# High-field magnetic resonance microscopy of aortic plaques in a mouse model of atherosclerosis

**DOI:** 10.1007/s10334-023-01102-1

**Published:** 2023-07-08

**Authors:** Rita Castro, Sean Gullette, Courtney Whalen, Floyd J. Mattie, Ximing Ge, A. Catharine Ross, Thomas Neuberger

**Affiliations:** 1 Department of Nutritional Sciences, Penn State University, PA 16802 University Park, USA; 2 Faculty of Pharmacy, Universidade de Lisboa, Lisbon, Portugal; 3 Huck Institutes of The Life Sciences, Penn State University, PA 16802 University Park, USA; 4 Department of Biomedical Engineering, Penn State University, PA 16802 University Park, USA

**Keywords:** Atherosclerotic plaque, Ex-vivo MRI, MR microscopy, Plaque burden

## Abstract

**Objectives:**

Pre-clinical models of human atherosclerosis are extensively used; however, traditional histological methods do not allow for a holistic view of vascular lesions. We describe an ex-vivo, high-resolution MRI method that allows the 3 dimensional imaging of the vessel for aortic plaque visualization and quantification.

**Materials and methods:**

Aortas from apolipoprotein-E-deficient (*apoE*^*−/−*^) mice fed an atherogenic diet (group 1) or a control diet (group 2) were subjected to 14 T MR imaging using a 3D gradient echo sequence. The obtained data sets were reconstructed (Matlab), segmented, and analyzed (Avizo). The aortas were further sectioned and subjected to traditional histological analysis (Oil-Red O and hematoxylin staining) for comparison.

**Results:**

A resolution up to 15 × 10x10 μm^3^ revealed that plaque burden (mm^3^) was significantly (*p* < 0.05) higher in group 1 (0.41 ± 0.25, *n* = 4) than in group 2 (0.01 ± 0.01, *n* = 3). The achieved resolution provided similar detail on the plaque and the vessel wall morphology compared with histology. Digital image segmentation of the aorta's lumen, plaque, and wall offered three-dimensional visualizations of the entire, intact aortas.

**Discussion:**

14 T MR microscopy provided histology-like details of pathologically relevant vascular lesions. This work may provide the path research needs to take to enable plaque characterization in clinical applications.

## Background

Vascular disease is the dominant cause of morbidity and mortality in the United States [1]. The most common cause of vascular disease is atherosclerosis, characterized by the accumulation of lipid-rich plaque inside the vessels. Pre-clinical models of atherosclerosis are valuable tools to advance understanding of the pathobiology of the disease. Apolipoprotein-E-deficient (*apoE*^−/−^) mice are an extensively used model of human atherosclerosis [2]. These mice display poor lipoprotein clearance with subsequent accumulation of cholesterol-ester-enriched particles in the blood, promoting atherosclerotic plaque development. The quantification of the amount of plaque in the aorta is used to assess the severity of atherosclerosis.

Traditional methods for probing aortic atherosclerosis in the *apoE*^−/−^ mouse model are mainly serial sectioning followed by standard histological analysis [3, 4], or en-face preparation followed by staining for lipids [5, 6]. In the first case, after serial sectioning, plaque areas are quantitatively segmented in only a subset of the sectioned slices. Moreover, some slices are damaged during the sectioning and/or the following preparation onto microscope slides. Further loss occurs due to the handling of the slides during lipid staining. Overall, serial sectioning is a time-consuming method that will result, due to the consideration of only two-dimensional slices, in an approximate volume estimate of the existing plaques. For *en-face* preparation, the isolated aorta is opened longitudinally, and the exposed lipid-rich plaques are subsequently stained with a lipophilic dye [7] allowing for a two-dimensional plaque burden analysis. Overall, these 2D serial sections methods present several limitations. For example, the scoring of the atherogenic phenotype relies on subjective measurements of a few representatives from different vessel regions, because not every section is stained and quantified. Moreover, parameters such as lesion topology, total plaque volume, and the location of the lesions in the vessel cannot be assessed. Finally, traditional histological analysis methods can often damage or distort plaques before 2D or 3D visualization. Using the fluorescence properties of the lipophilic dye Sudan IV, e.g., Jung et al. [7] were able to determine plaque volume in *en-face* prepared aortas using confocal fluorescence microscopy. They were able to report the progression of plaque burden in a 3D fashion. Due to the low penetration depth of confocal fluorescence microscopy, an *en-face* preparation was still required.

An alternative methodological approach to visualize and quantify the aortic plaque in intact aortas of *apoE*^−/−^ mice involves the use of magnetic resonance imaging (MRI) [7–9]. The soft tissues of the cardiovascular system are ideal candidates for MRI, since they can be distinguished by their unique MRI properties [10]. Furthermore, MRI offers multiple advantages over the traditional methodological approaches mentioned above. Notably, MRI allows for the preservation of the aortic tissue allowing subsequent experimentation while providing 3D imaging, which is amenable to digital reconstruction for further analysis and presentation. MRI has been previously used to quantify aortic plaque volume from the aortic root to the descending aorta and in specific regions of the vessel, including the highly susceptible brachiocephalic artery [6, 11–14]. Qualitative analysis of atheromatous plaque in *apoE*^−/−^ mice by MRI has also been achieved [6, 7, 11, 12, 15, 16], but the resolution remains inferior to that obtained with traditional histological analysis. Advancements in this area would be highly valuable, since the risk of cardiovascular events is associated with specific characteristics of the lesions, such as fibrous cap disruption or the presence of a lipid-rich necrotic core [17–19].

Here we describe an ex-vivo, high-resolution MRI method that allows three-dimensional imaging of the intact aorta for plaque quantification and visualization of histology-like details.

## Methods

### Animals

Seven-week-old *apoE*^−/−^ mice were purchased from Jackson Laboratory (Bar Harbor, ME, USA) and housed in a temperature- and humidity-controlled room. Only male mice were used to control for the known effect of gender on atherosclerosis in this strain [20]. The animals had free access to water and to a control diet (5% w/w fat) or to an atherogenic, high-fat diet (20% w/w fat and 0.15% cholesterol). Diets were prepared based on AIN 93G (Research Diets, New Brunswick, NJ, USA) and were previously described [21, 22]. All procedures were performed in compliance with the Institutional Animal Care and Use Committee of the Pennsylvania State University, which specifically approved this study.

### Ex vivo sample preparation

**Fig. 1:**
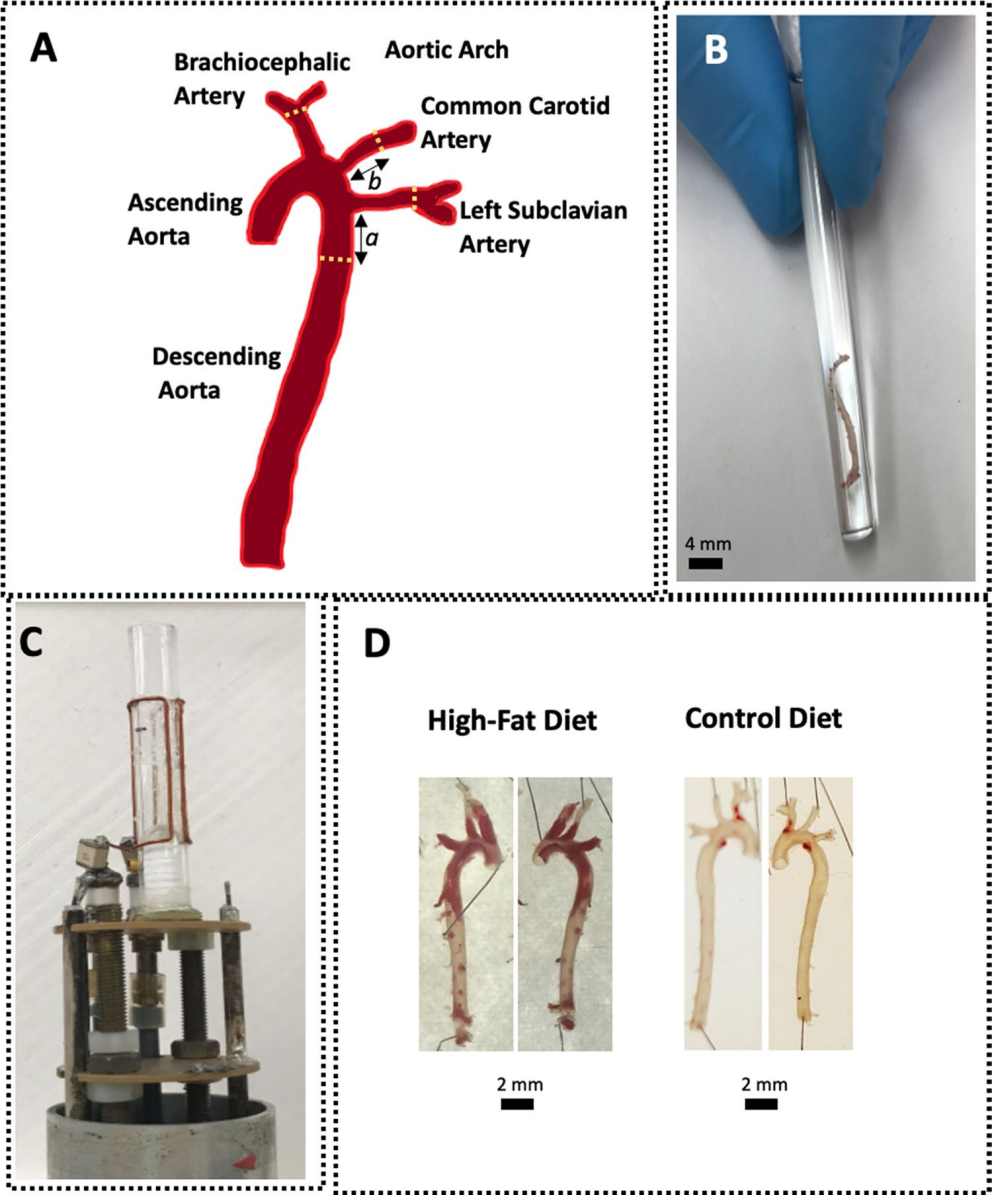
**A** Illustration of the anatomical regions of a mouse aorta. The dashed white lines denote limits used for the volumetric quantification; a denotes the length of 400 slices, b denotes the length of the shortest common carotid artery in the batch of aortas that were imaged (see text for details) **B** One aorta in the glass tube prepared for MRI. **C** Home-made saddle coil. **D** Representative example of modified e*n-face* preparation of one aorta from high-fat diet or control*-*fed* apoE* ^−/−^ mice, stained for lipids with Oil Red O. Plaque appears red and both sides of the aortas are shown

After 12 weeks on each diet, the mice were euthanized by carbon dioxide inhalation. After the exposure of the aorta, 10 mL of cold phosphate saline buffer (PBS) were perfused using a syringe through the left ventricle of the heart. The aorta was then slowly (1 mL/min) perfused through the heart in situ via the left ventricle with 5 ml of 10% neutral buffered formalin (NBF) in PBS and excised along with the heart. Particular care was taken to ensure that the branches of the aortic arch (brachiocephalic, carotid, and subclavian arteries; Fig. 1A) were intact. The aortic tissue, heart, and associated surrounding tissue were subsequently fixed in 10% NBF overnight and washed with PBS. To facilitate complete adventitial fat removal, the tissues were subjected to Oil Red O staining prior to fine dissection, as previously described in detail [21–24]. Aortas were again Oil Red O stained to enhance plaque visualization, pinned out intact, and photographed on each side. Subsequently, aortas were equilibrated in a solution of 0.1% Magnevist (Bayer, Whippany, NJ, USA), 0.25% sodium azide in PBS overnight at 4 °C, re-equilibrated to room temperature. The aortas were placed into glass culture tubes (6 mm OD, 4 mm ID and 60 mm length; Fig. 1B). After inserting the aortas into the tubes, most were very well-held due to the tube dimensions and did not move. Fixing the aortas and the associated stiffening of the tissue might have helped keeping them in place as well. However, when the aorta was too short and was sliding to the bottom of the tube, cotton was placed into the bottom of the tube before the aorta was inserted again. This ‘cotton bed’ was enough to hold the aortas in place. Furthermore, it kept them away from the bottom of the tube preventing artifacts that could occur when specimens are placed too close to the bottom of a tube. To prevent the whole glass tube from moving, a toothpick (wedged in between the glass tube and the RF-resonator) and parafilm were used at the top to secure the glass tube.

To reduce the risk of bubbles we used degassed PBS and prepared the Magnevist solution a day before immersing the aorta to ensure no bubbles formed in the solution. After transferring the aortas to the glass tubes, we used a surgical microscope to check for visible bubbles. If bubbles were detected, we removed them by either gently tabbing the glass tube onto the counter top or by extracting them with a syringe with a blunt tip. To ensure that no bubbles have formed inside the aorta we ran two-dimensional low-resolution coronal gradient echo images (echo time 2.6 ms, in plane resolution: 78 × 52 μm^2^, slice thickness: 1 mm) before the long three-dimensional scans. If dark spots were visible in these low resolution images, another search for bubbles started. In very rare cases bubbles were trapped inside the aorta. To remove these bubbles, aortas were gently flushed with the PBS solution using a pipet. After ensuring that no bubbles were present, the samples were subjected for MRI analysis.

### Imaging

MR microscopy was performed on an Agilent 14 T micro-imaging system using a home-built saddle coil with an inner diameter of 7 mm. Standard 3D gradient echo imaging was performed at two different resolutions. A high-resolution set: TE = 11 ms, TR = 140 ms, flip angle 60°, 8 averages, FOV of 7.5 × 4.0 × 4.0 mm^3^, matrix size of 500 X 400 X 400 (resolutio* n* = 15 X 10 X 10 µm^3^) and scan times of approximately 50 h; and a lower-resolution set: TE = 10 ms, TR = 100 ms, flip angle 40°, 8 averages, FOV of 12.6 × 4.2 × 4.2 mm^3^, matrix size of 630 × 210 × 210 (resolutio* n* = 20 × 20 × 20 µm^3^) and scan times of approximately 10 h. After zero-filling by a factor of two during the reconstruction with Matlab (The MathWorks Inc., Natick, MA), the final image pixel size, not the actual resolution, was 7.5 × 5 × 5 µm^3^ and 10 × 10 × 10 µm^3,^ respectively. The higher resolution set was obtained to facilitate the visual analysis of plaque composition, while both sets were used for segmentation and quantification.

### Segmentation and quantification

Data segmentation and quantification was performed using Avizo (Thermo Fisher Scientific, Waltham, MA) for MRI analysis. Aortas were partitioned into three segments: lumen, plaques, and wall. Avizo’s lasso tool and interpolation function were utilized for gross segmentation and the brush tool was utilized for finer details. The total plaque volume was obtained using the material statistics function under the project view in Avizo.

Only the arch and its three branches were considered in the quantitative analysis. The arch was defined as the section of the aorta between the aortic valve and the descending aorta (400 slices after the left subclavian artery. Furthermore, the brachiocephalic artery and the subclavian artery were digitally truncated at their respective points of bifurcation. The carotid arteries were digitally truncated at a point equal to the shortest carotid length (see Fig. 1A). Two independent researchers segmented the truncated aortas to account for inter operator variability.

Plaques were identified by their morphological and contrast differences from the aortic wall. Specifically, plaques could be seen to bulge from the ordinarily cylindrical walls and would often have an irregular border. In addition, plaques often had a higher gray-value intensity than the wall tissue, and when advanced far enough, could be seen to contain much lower-intensity components, such as lipid pools which are not found in aortic walls.

### Aorta cryosectioning and histological analysis

Following MRI analysis, aortas were equilibrated in sucrose solution as a cryoprotectant, as previously described [22]. Aortas were then pinned out into arrays, embedded in Tissue-Tek® Optimal Cutting Temperature compound (OCT, Sakura® Finetek, Torrance, CA, USA), and stored at − 20 °C for 24 h. The array blocks were then sectioned perpendicularly to the vessel into 12 μm thick sections using a Leica CM3050 S cryostat, at − 20 °C and attached to TruBond™ 380 microscope slides (Electron Microscopy Sciences, Hatfield, PA, USA). Slides were dried at least 1 h at 32 °C, and kept at − 20 °C until hematoxylin staining, in which Mayer’s hematoxylin solution (Sigma-Aldrich, Cat. #MHS16, St. Louis, MO, USA) was used as previously described in detail [22]. Slides were processed according to the manufacturer’s instructions, using a 5 min. incubation time, then mounted in an aqueous mounting medium (VECTASHIELD® Vibrance™ Antifade Mounting Medium, Vector Laboratories, Burlingame, CA, USA). Oil Red O remained in tissue through the sectioning process and did not require re-application.

### Statistical analysis

Analyses were performed in GraphPad Prism 7 (GraphPad Software, La Jolla, CA, USA), with statistical significance set to *p* < 0.05. An unpaired Student’s t test was used to compare the plaque volume between the two groups of mice, after the normal distribution of the data was confirmed using the Shapiro–Wilk test.

## Results

Modified *en-face* visualization of aortas allowed a semi-quantitative assessment of plaque burden and revealed a profound difference between the two groups of animals (Fig. 1D).

**Fig. 2:**
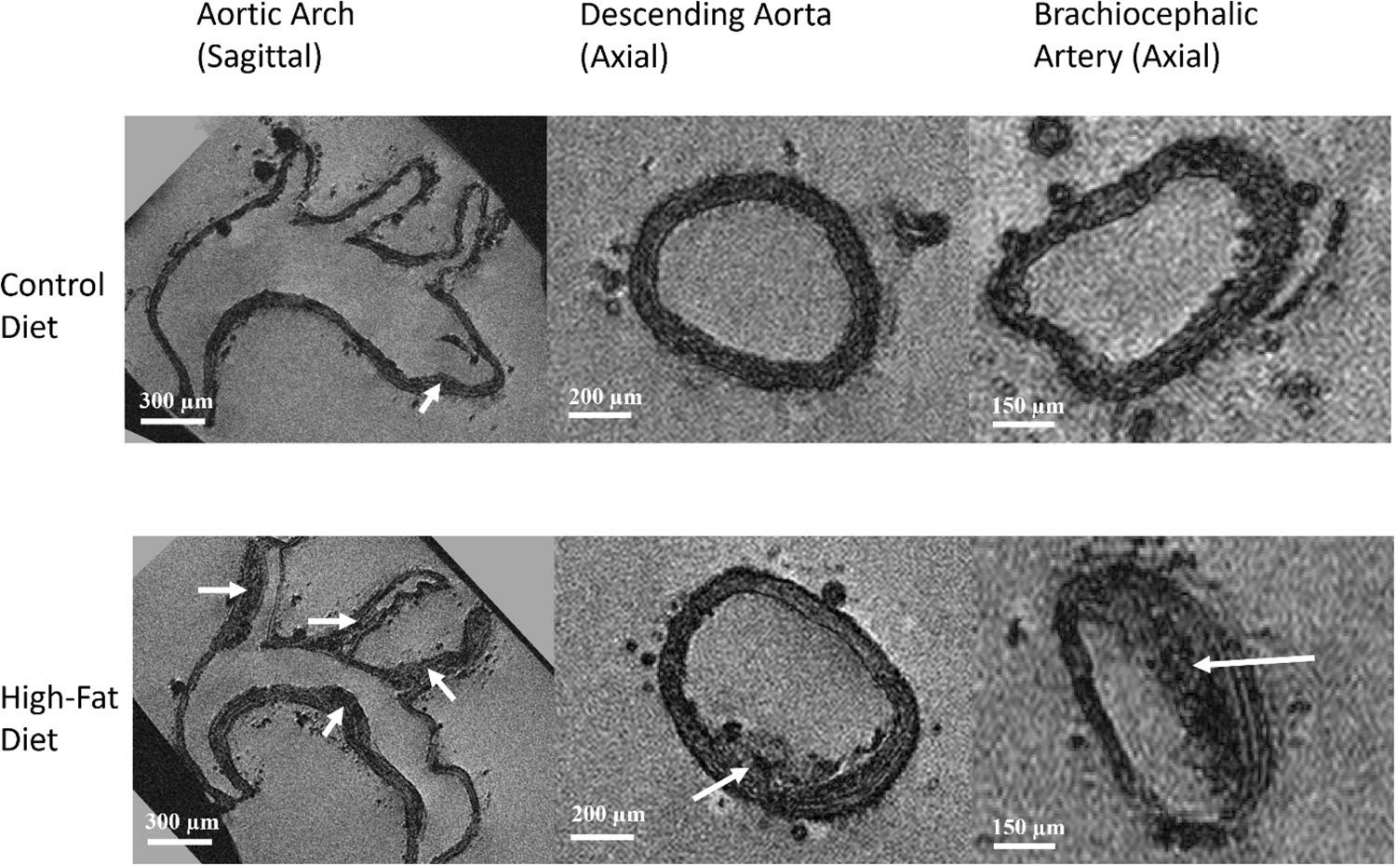
MR microscopy of aortas. Representative images of the control-diet and high-fat-diet groups of the higher resolution data sets at different aortic segments, with white arrows denoting the plaque

Representative high-resolution MR microscopy images of aortas from the high-fat-diet group and the control-diet group are shown in Fig. 2. As expected, there is a clear difference in the aortic plaque burden in the aortas from both groups, with the high-fat diet fed mice presenting larger, and more numerous plaques than the control-diet fed group.

**Fig. 3:**
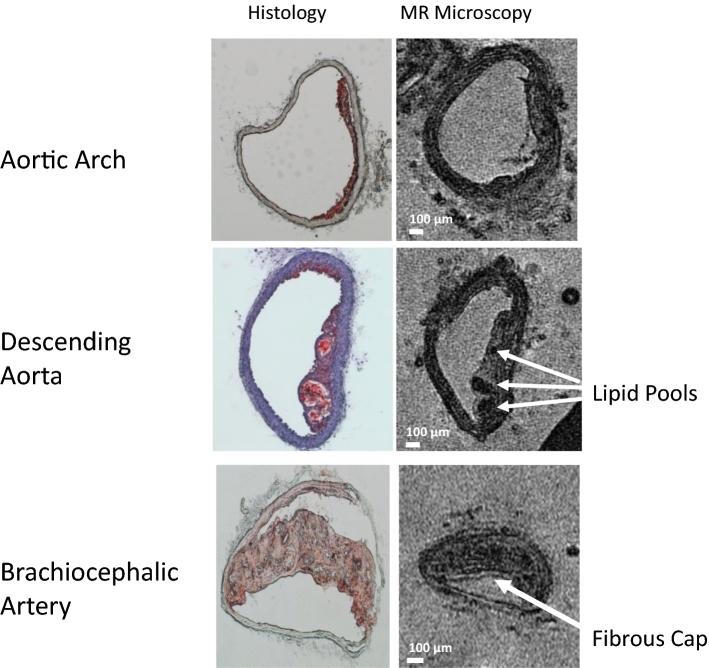
Comparison of MR images of mice aortas and their equivalent histology sections from the aortic arch (top), the descending aorta (middle), and the brachiocephalic artery (bottom). All MR images are from higher resolution data sets

Subsequently, stained cryosections of the same aortas were compared with images from the MR data set. Various features, such as plaque and vessel morphology, were used to identify similar locations for juxtaposition of the images. Figure 3 displays some of these comparisons, and several plaque characteristics can be observed in the images. For example, the top images (aortic arch) show a thin plaque (≈70 μm thickness). In the middle images (descending aorta) a more mature plaque (≈150 μm thickness) containing multiple lipid pools is shown. The bottom images (brachiocephalic artery) show the most advanced lesion (≈200 μm thickness) displaying a fibrous cap and diffuse microcalcifications. Notably, many of the larger plaques were intact during MRI but became detached from the vessel wall during the sectioning process; the wall itself was often damaged during histology.

**Fig. 4:**
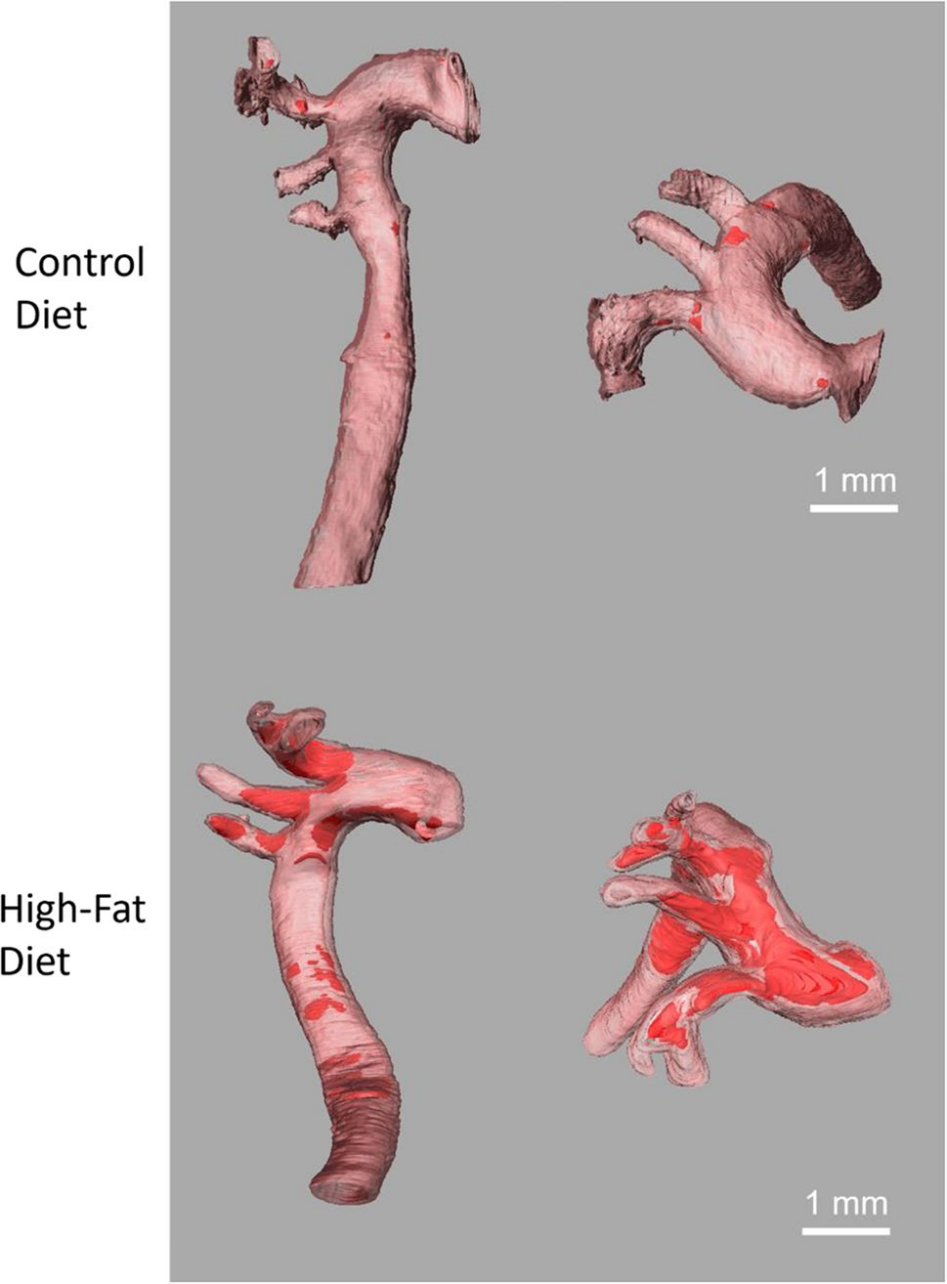
Representative segmentations of the thoracic aorta from a control diet sample and a high-fat-diet sample. The vessel wall (pink) is set to a moderate transparency to allow visualization of the plaques (red) inside. Side views (left) and top views (right) of the aortas are shown

After the MR data set was reconstructed and segmented, a surface view was created to visualize the overall aortic plaque distribution. Representative surface views of the entire thoracic aorta from a high-fat-diet and a control-diet fed mouse are shown in Fig. 4. The vessel wall has been set to a moderate transparency level to emphasize plaque visualization. Atherosclerotic plaques are shown in red. As expected, larger, and more abundant plaques were observed in the aortas from the mice fed with the high-fat diet.

**Fig. 5:**
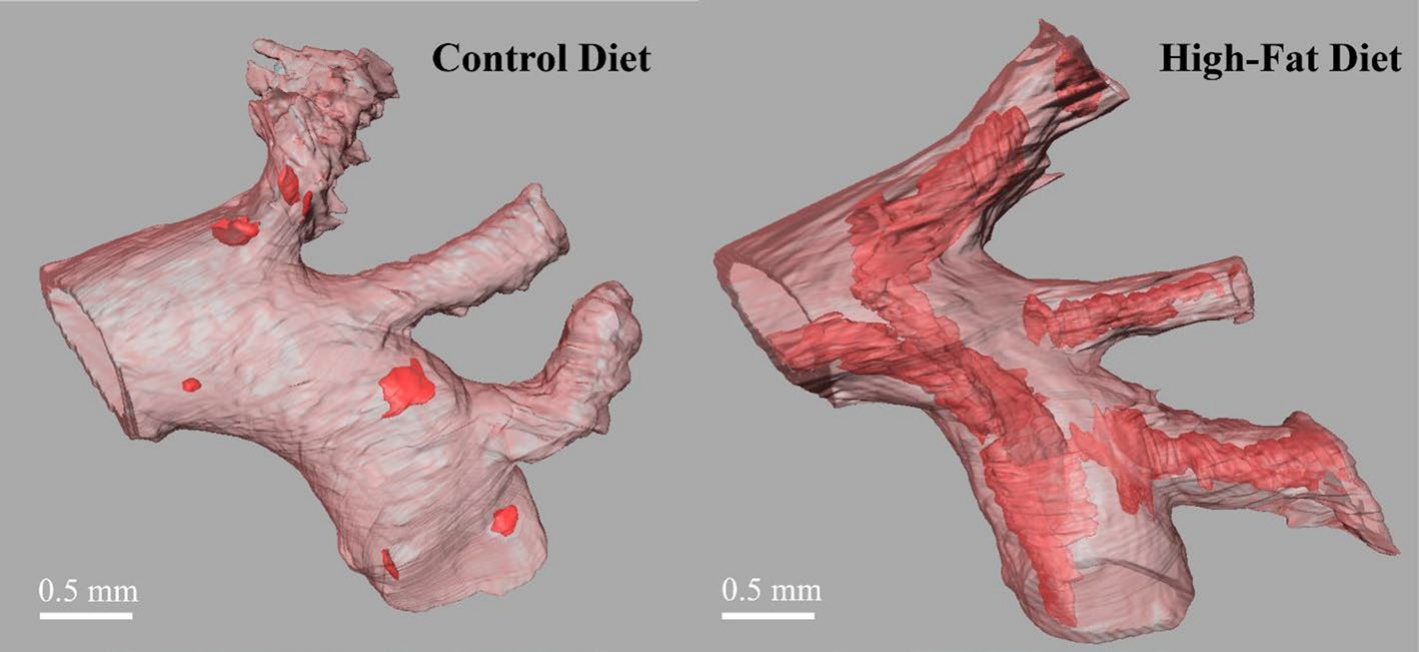
Representative image of the aortic arch and three branches used for volumetric quantification

To standardize the image analysis, aortas were digitally resected, leaving the aortic arch and its three main branches for quantification of the lumen, plaque, and vessel wall (see Fig. 5). Each carotid artery was truncated to match the length of the smallest ($\approx 1.1mm$). The brachiocephalic arteries and the subclavian arteries were truncated at their bifurcations.

**Fig. 6:**
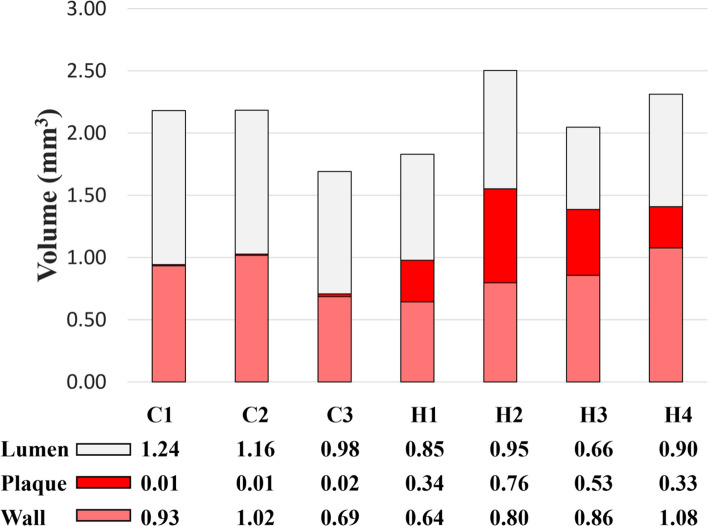
MRI quantification of the lumen, plaque, and wall of mice fed a control diet (C1–C3) or a high-fat diet (H1–H4)

The total volumes of the segmented lumens, plaques, and walls of the mouse aortas are shown in Fig. 6. The plaque volume (mm^3^) ranges from 0.33 to 0.76, and 0.01 to 0.02, in the high-fat-fed mice and controls, respectively. In accordance with the phenotype shown in Figs. 2 and 4, a significantly (p = 0.0105) greater plaque volume (mm^3^; mean ± STD) was observed in the high-fat-diet fed group (0.49 ± 0.20, *n* = 4) than in controls (0.013 ± 0.006, *n* = 3).

**Fig. 7:**
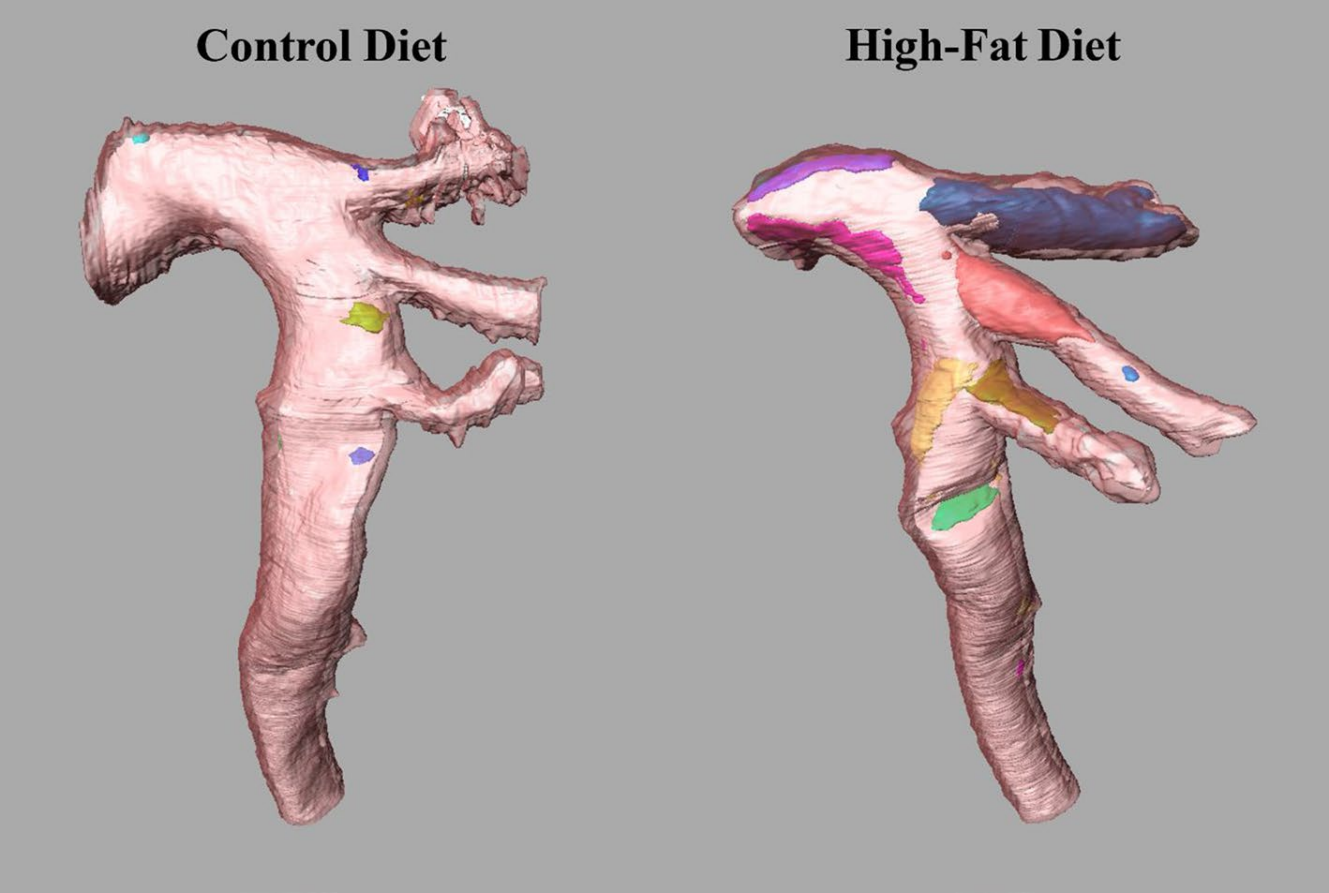
Individually segmented plaques in a control and a high-fat-diet fed group. Different colors were used to represent each plaque

As shown in Fig. 7, the individual plaques can be identified, and localized to identify the regions more susceptible to developing atherosclerosis. In accordance with previous reports [21–24], the plaque is concentrated in the more susceptible regions: brachiocephalic, carotid, subclavian arteries and in the inferior curvature of the aortic arch. Assessment of the individual plaque volume was performed. For example, in the aortas shown in Fig. 7, the individual plaque volume (mm^3^) ranged from 0.002 to 0.02 in the control-diet fed mouse and from 0.001 to 0.3 in the high-fat-diet fed animal. Interestingly, the number of plaques found in both aortas was approximately the same (10 and 11, respectively); however, the sizes were drastically different, suggesting that smaller plaques in the high-fat-diet fed mice have merged [25].

## Discussion

For the first time, we present detailed methods for applying 14 T MR microscopy ex vivo to characterize the phenotype of the aorta of a pre-clinical mouse model of atherosclerosis. Previous studies, in the same mouse model, applied 17.6 T MR microscopy [26] or 11.7 T MR imaging [6] to characterize the phenotype of the atherosclerotic aorta but this is, to the best of our knowledge the first time such ex vivo studies were conducted at 14 T yielding histology-like images. The difference of aortic plaque volume between the high-fat-diet group and control-diet group was significant. The ability to image the entire thoracic aorta enabled the rendering of visually expository reconstructions which provide information about plaque volume, position, and topology.

Qualitatively, the 7.5 × 5 × 5 µm^3^ display resolution enabled the visualization of pathologically relevant lesion characteristics, such as fibrous caps and lipid-rich pools. Such details could improve the analysis and determination of disease severity in atherosclerosis [27].

The number of animals used in this study was small (control diet: *n* = 3; high-fat diet: *n* = 4), making intragroup statistical inference weak. Nevertheless, we were able to detect a significant difference in the plaque burden between the two groups.

While spin echo sequences are known to have less distortions/signal loss due to their inherent T2 weighting compared to gradient echo sequences, three-dimensional gradient echo imaging has been used successfully elsewhere to obtain high resolution images of mouse embryos ex vivo[28]. In the presented study the addition of Magnevist reduced the T1 time significantly and, therefore, allowed despite a relative short repetition time (100 ms or 140 ms) flip angles of 60 degrees resulting in a relative high SNR. As T2 times of tissues are already relatively short and keeping in mind that the used Magnevist concentrations might have shortened these T2 times even further, a spin echo-based sequence was not a viable option. We tested a standard three-dimensional spin echo sequence with similar resolution and ended up getting a very low SNR despite a longer acquisition time (data not shown). Using shorter repetition times in our work might have helped boosting the SNR even further. Unfortunately, due to gradient duty cycle limitations on our system this could not be realized. With an in plane resolution of 10 microns we are close to the diffusion limit of MR microscopy, increasing the used echo times would have resulted in significant signal loss. Reducing the echo times was not possible due to gradient strength limitations.

Manual image segmentation is an inherently subjective and time-consuming process. Importantly, however, a framework for automated 3D segmentation of vessel wall and quantification of atherosclerotic plaque has already been developed [29]. Such automated methods not only streamline the time-consuming process of manual segmentation but also increase the objectivity of the process by eliminating the human subjectivity. Previous research has shown the ability of MRI to differentiate between various components of plaque [30]. For example, Merickel et. al. used pattern recognition to differentiate between calcified plaque, complex fibrous plaque, and fatty plaque [31].

During the optimization of this method additional experiments were conducted to achieve a hyperintense signal for the lipid cores. Chemical shift selective imaging was applied to image the lipid distribution within the aorta. To ensure that the relative high concentration of Magnevist, the staining, and the fixation did not affect the lipid signal, we did these experiments on fresh unfixed and unstained aortas. Unfortunately, the applied chemical shift selective imaging was not successful (data not shown). Specifically, a frequency band nullification was applied to the water signal to acquire a lipid-only image. However, only adventitial fat (outside of the blood vessel) was detected and no intraplaque lipids were visible. Reasons for this are uncertain, but it is possible that the lipid concentration was too low and/or the T2* of atheromatous lipids at 14 T are relatively short compared to the T2* of adventitial lipids. A previous study estimated a T2* for human atheromatous lipids (at 11.7 T) of 6 ms [32]. Despite conducting these experiments without the addition of Magnevist, which could shorten the T2* at the used concentration, and using a relatively short echo time (2.8 ms) no atheromatous lipids could be visualized. Further studies need to be conducted to investigate these results.

The upper mouse aorta is about 1 mm in diameter, the human upper aorta is about 2.5–3 cm [33]. 20 micron resolution was used in this work in the mouse which is about 50 pixels across the aorta. For a human aorta with 50 pixels across, we would need a resolution of 3 cm/50 = 0.6 mm, which is a resolution that is already possible for higher field human scanners. The imaging time for the presented segmented data was about 10 h in the mouse. Using less averaging, a resolution of 0.6 mm, and saturation bands (smaller FOV), imaging times of a few minutes might be achieved making this method not too far away from being used in the clinic.

## Conclusion

High-field MR microscopy was applied to visualize and quantify plaque burden in a mouse model of atherosclerosis. Histology-like detail of pathologically relevant lesion characteristics was attained. This work may provide the path research needs to take to enable plaque characterization in clinical applications.

## Abbreviations

MRI: Magnetic resonance imaging
apoE^−/−^: Apolipoprotein-E-deficient
PBS: Phosphate saline buffer
NBF: Neutral buffered formalin
OCT: Optimal cutting temperature
